# Darwinian rebel: The legacy of Loh-Seng Tsai—Chinese animal psychologist

**DOI:** 10.1007/s13238-021-00849-4

**Published:** 2021-05-26

**Authors:** Yong Wang, Wei Chen, Shiying Li, Bin Yin

**Affiliations:** 1 Center for Brain, Mind, and Education, Shaoxing University, Shaoxing, 312000 China; 2 School of Psychology, Fujian Normal University, Fuzhou, 350108 China

**Figure 1:**
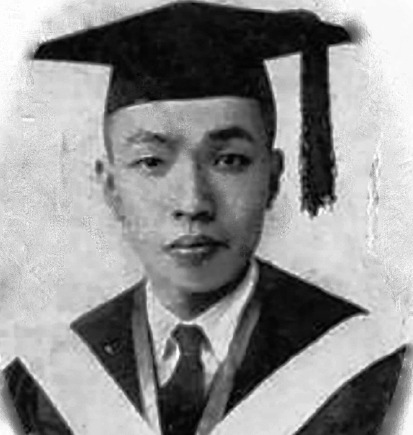
Loh-Seng Tsai in a bachelor’s gown in 1923

Loh-Seng Tsai (蔡乐生, 1901**–**1992) (Fig. 1), the world-renowned Chinese animal psychologist and psychopharmacologist, was born to a merchant family in Chaoan County, Guangdong Province on February 6th, 1901. He was nominated for the Nobel Peace Prize in 1951 and U.S. President Nixon praised his “exemplary academic achievements” in 1973.

Tsai’s research in experimental psychology can be divided into two stages.

During the first stage (1919**–**1949), Tsai embarked on the path of psychology as a student under the guidance of Clarence Herbert Hamilton. Subsequently, Tsai expanded and popularized the experimental research conducted by comparative psychologist Zing-Yang Kuo (郭任远, 1898**–**1970) and Harvey Carr, both of whom were Tsai’s supervisors at different times.

In the summer of 1919, Tsai attended Nanking University to study psychology following the footsteps of Dr. Hamilton, who had obtained his Ph.D. in philosophy from the University of Chicago, later becoming a professor of philosophy and psychology, finally chairing the Philosophy Department until the end of his tenure at Nanking University in 1927. Among the many psychological instruments he brought from the USA was the unpublished “Textbook of Experimental Psychology” manual (compiled by Woodworth and Poffenberger), which he used to teach students how to conduct experiments. It represented the most cutting-edge knowledge of Western psychology that Chinese students encountered in the early 1920s. It had a profound impact on Tsai and prompted him to pursue a career in psychology (Yan, 2015).

After graduating from Nanking in 1923, Tsai served as a psychology assistant to Dr. Zing-Yang Kuo at Fudan University. At that time, the School of Psychology of Fudan University had been growing rapidly under the leadership of Dr. Kuo, the “Out-Watsons Mr. Watson” behaviorist. He introduced the American-inspired anti-instinct movement in psychology to China in the late 1920s and prompted a major debate on the issue of “instinct” in domestic academic circles, which would later become one of the three major polemics in the history of modern psychology in China (Zhang, 1983; Yan, 1998; Che, 2004). Notably, Dr. Kuo’s cat-rat cooperation experiment denied the validity of instincts with experimental evidence for the first time, leading Tsai to realize the power of laboratory work. While teaching applied psychology courses such as experimental, business and forensic psychology during his time at Fudan University, Tsai was also engaged in experimental research. The result, his first experimental article “*The relation of retention to the distribution of relearning*”, was published in *The Journal of Experimental Psychology* (Tsai, 1927). During that time, Tsai was awarded a Master of Science degree by Fudan University on the basis of this article from 1926, becoming the first master’s degree student in psychology trained in China (Zhou, 2015). Interestingly, this article had been cited 28 times according to Google Scholar, most recently in 2020.

Tsai studied at Chicago University in July 1926, engaging in experimental research on animal psychology under the supervision of Harvey Carr, the late representative of the Chicago school of functional psychology. Tsai’s doctoral thesis entitled “*Gradual versus abrupt withdrawal of guidance in maze learning*” was completed in 1928 and it was subsequently published in *The Journal of Comparative Psychology* (Tsai, 1930a). The study, addressing the effects of two different instructional methods in maze learning experiments with rats, proved that repeated instructions could inhibit individual learning, which fundamentally challenged the relationship between teaching and learning. The result was cited by Tolman (1938) to overthrow the law of exercise—proposed by the well-respected educator and psychologist, Thorndike, who considered that any stimulus-response combination will gradually be strengthened during trial and error learning through regular practice and application. In addition, Tsai (1931) discovered the cul-de-sac phenomenon in an experiment, shedding light on the mechanism of turns, which is still influential in present-day comparative psychology experiments concerning maze problems, simple or multiple choice problems, and similar scenarios.

Following a recommendation by Professor Carr, Tsai visited the Departments of Pathology and Psychology at the University of Chicago as a researcher and associate professor in 1928, where he engaged in research on the effects of hormones on the development of the individual nervous system. Through a comparative experiment on castrated rats, he found that sex hormones can directly stimulate the nervous system to improve the rats’ social adaptation. Subsequently, Tsai presented a report entitled “*Sex glands and adaptive ability*” at the Ninth-International Congress of Psychology in 1929, which was supported by Pavlov’s report (Tsai, 1930b). Concurrently, while other scholars were still studying the function of vitamin B, Tsai and his colleague Maurer published the pioneering article “*Vitamin B deficiency in nursing young rats and learning ability*” in *Science* on the effects of vitamin B deficiency on the learning, memory, and problem-solving abilities of rats (Maurer & Tsai, 1929). They found that insufficient vitamin B-complex supply during pregnancy and lactation affected the development of higher neurological functions in young rats, inhibiting their intellectual development. They correlated these findings with the anatomical and chemical changes in the nervous system and presented a novel way to explore the relationship between the nervous system and learning ability (Maurer & Tsai, 1930). On this basis, Tsai and Maurer studied the intergenerational genetic effects of vitamin B-complex deficiency in rats and found that the adverse effect on learning ability does not pass through the germ plasm to the offspring (Maurer & Tsai, 1931). These experimental findings later were confirmed by other scholars (Bernhardt, 1934; Poe et al., 1936). At the same time, Tsai also focused on the genetic problems of individual behavior and accidentally discovered that rats also have a right-handedness tendency, similar to that of humans. Further systematic experimentation confirmed that normal rats—regardless of gender—were mostly right-handed (Tsai & Maurer, 1930). This discovery contributed to the controversial “human right-handedness” discussion and shed light on the phylogenetic evolution of “handedness”. In addition, it challenged the theories of the outgrowth of human intelligence, primitive warfare, and social trajectory. Interestingly, Tsai also found that most of the rats lacking vitamin B were left-handed (Tsai & Maurer, 1930).

Upon receiving an invitation from the Academia Sinica, Tsai returned to China in July 1931 to work as a researcher at the National Research Institute of Psychology. Here, he carried out a series of experimental studies on the memory curve of rats, delayed reaction, and animal comprehension. His greatest influence was related to two laws of animal behavior. The first of these is the “minimum effort”, and the second law is “maximum satisfaction” (Tsai, 1932). To prove them, Tsai used twenty-one experiments: eight conducted according to literature by Zing-Yang Kuo, Tolman, and others to prove the laws, and the remainder coming from his own work. These experiments were clustered into eight series of tests and allocated to a “law” for minimum effort (1 = relative weight of resistance; 2 = relative height of obstacle; 3 = relative width of pathway; 4 = relative length of alley; 5 = relative time of passage) and for maximum satisfaction (6 = degrees of relief; 7 = kinds of relief; 8 = summation of relief). Tsai believed that the two laws, especially maximum satisfaction, proved that behaviors are not linked to the number of repetitions, as Watson had emphasized, but the amount of satisfaction or number of rewards. This kind of satisfaction means has no subjective meaning, according to the needs of the organism, and is incomparable according to Thorndike’s law of effect (Academia Sinica, 1932).

**Figure 2:**
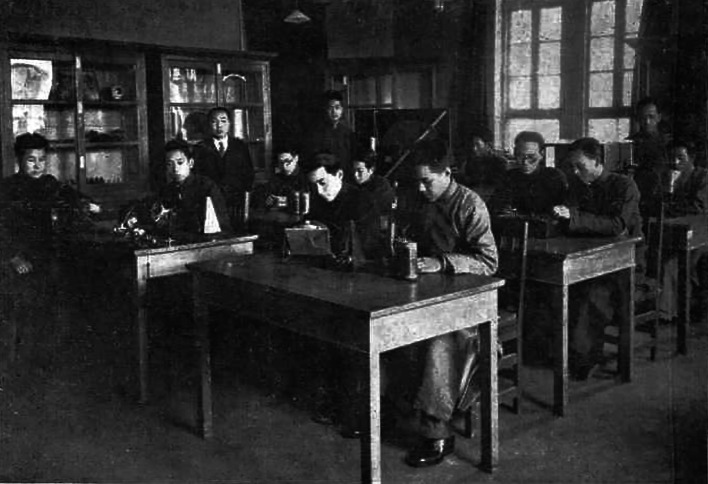
In 1936, Loh-Seng Tsai (third from left) instructed students at the National Peiping Normal University in conducting psychological experiments

**Figure 3:**
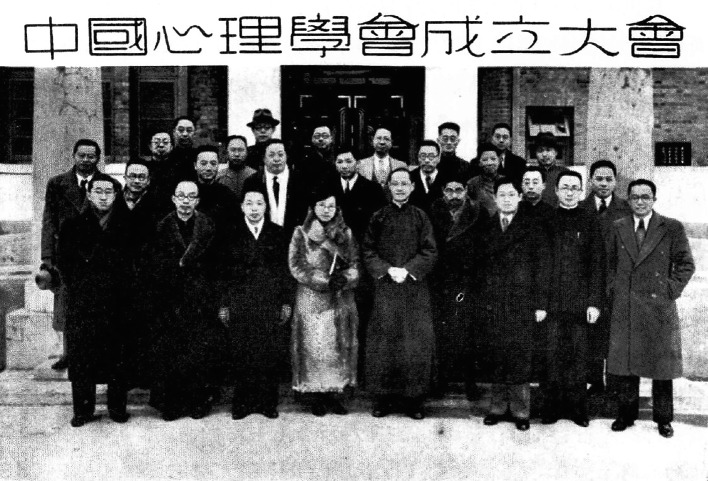
On January 24th, 1937, Loh-Seng Tsai (first row, third from left) attended the founding meeting of the Chinese Psychological Society

After Tsai obtained a professorship and founded the psychological laboratory at Henan University in 1933, he became the director of the Research Institute of Educational Psychology at National Sun Yat-sen University the following year. Thereafter, he lectured in the Department of Psychology at the National Beijing Normal University (Fig. 2). When Nanking University moved to Huaxiba, Chengdu, in 1937, he became the head of the Philosophy Department of the College of Arts. In that year, Tsai attended the founding meeting of the Chinese Psychological Society in the Nanjing National Compilation Hall, becoming one of 34 sponsors of the Chinese Psychological Society (Fig. 3). He proceeded to first become chairman of the newly established Department of Philosophical Psychology in 1940, and later dean of the Faculty of Arts at Nanking University in 1944. Three years later, the Normal College of Sun Yat-sen University sent Tsai to replace Itzen Kuo (郭一岑) as director of the Institute of Education and director of the Department of Psychology. Tsai settled in the United States in 1950 to teach at Tulane University while carrying out a series of creative animal behavior experiments.

**Figure 4:**
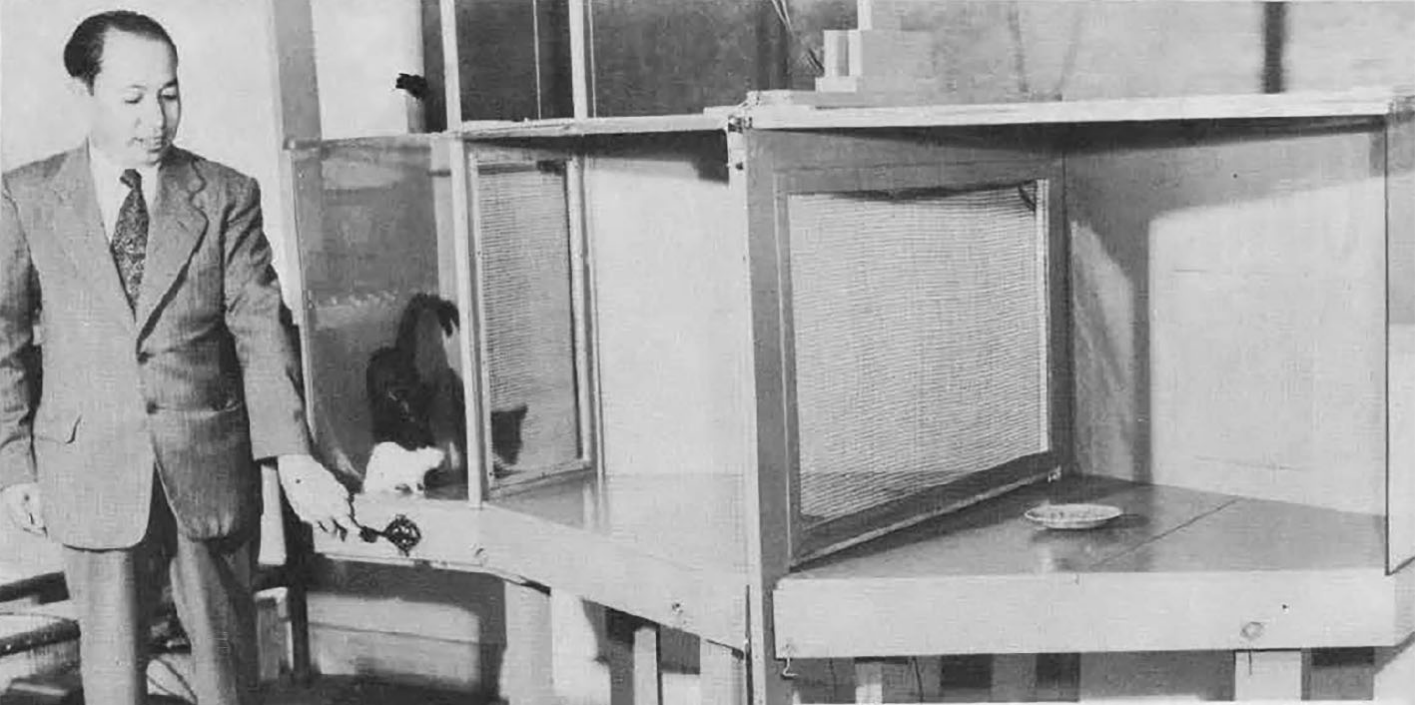
In 1951, Loh-Seng Tsai conducted experiments on cooperation between cats and rats in a specially designed box

During the second stage of Tsai’s career, starting in the 1950s, his interest spanned two main areas. First, deeply skeptical of Darwin’s theory of evolution and his views on survival of the fittest in an era dominated by Darwinism, Tsai (1950) proved the potential of rats to both compete and cooperate. In 1951, he designed a unique apparatus—“Tsai’s cooperation box” (Fig. 4)—in his laboratory at Tulane University. It consisted of three sections (entrance area, reaction chamber, and goal chamber) that were separated by electrically controlled screened gates. The cat and rat, who had been accustomed to cohabiting, were placed in the entrance area prior to the test. When the gates were opened, they entered the reaction chamber where they had to cooperate (both stepping on the floor buttons simultaneously) to access the food in the goal chamber. Through educational cooperation—without resorting to punishment—Tsai helped “natural enemies” to become amicable (Tsai, 1951). He believed that the decisive cat-rat cooperation experiment proved, for the first time, that there is neither a “pugnacity instinct” (McDougall, 1908), nor a “fighting instinct” (Tolman, 1942). Notably, Tsai’s research on peace and cooperation laid the biological foundation for the theoretical possibility of world peace—prompting his nomination for the Nobel Peace Prize in 1951. He stated that “the world today is in need of a new philosophy of life” and proposed his philosophy of “survival through cooperation” to replace Darwin’s “survival of the fittest” (Tsai, 1963), steering education toward world peace.

**Figure 5:**
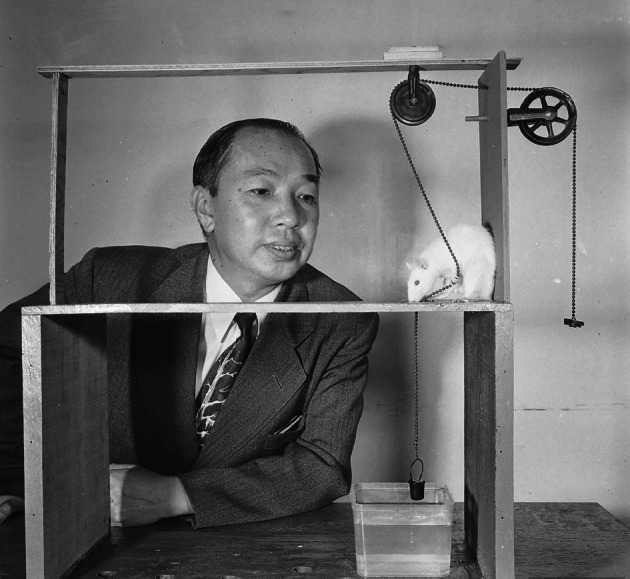
Loh-Seng Tsai watches one of his smart pupils haul up a bucket of water in 1952

**Figure 6:**
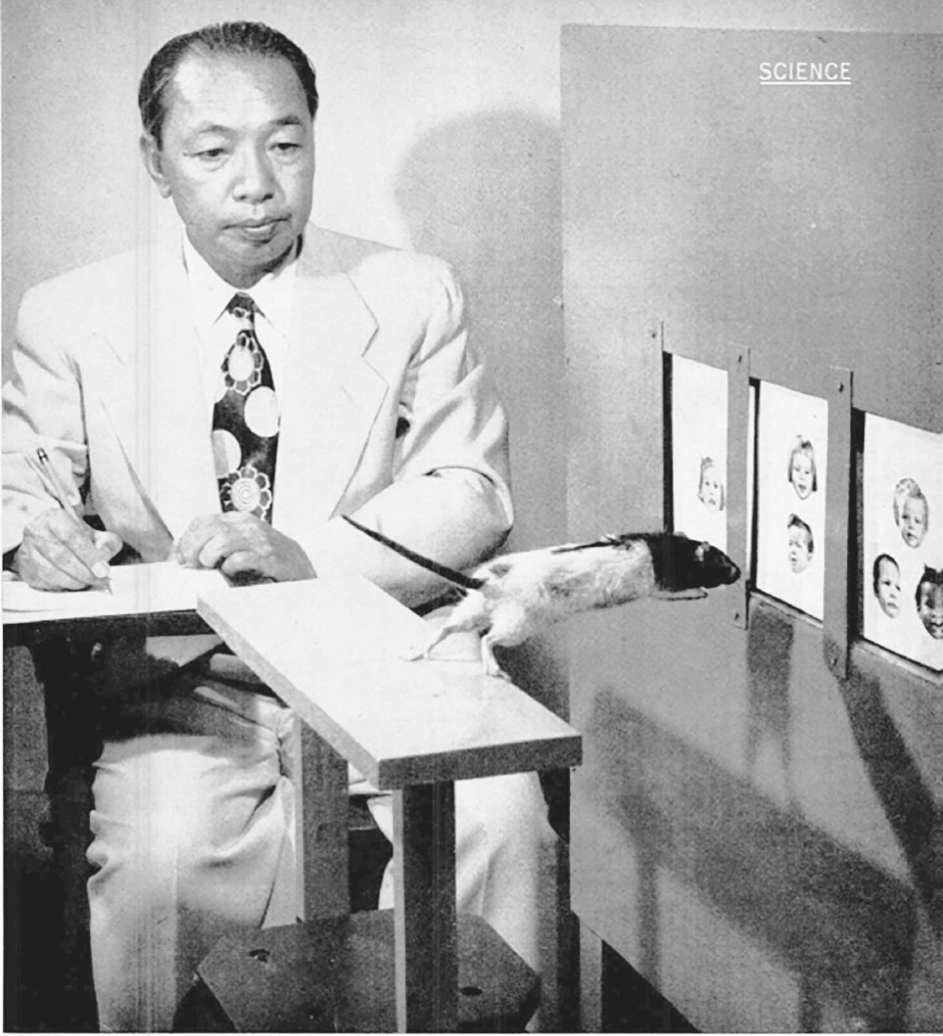
In this photograph from 1954, a rat trained by Loh-Seng Tsai is seen unhesitatingly leaping toward the door marked with two images, which leads him to his dinner

In addition, Tsai also remained engaged in experimental research on the socialization of animal behavior. *Life* magazine once commented: “Dr. Tsai’s experiments, fascinating in themselves, have a more serious purpose than simply to teach rats extraordinary tricks” (Tsai, 1952). In order to prove the rats’ reasoning ability, which most psychologists attributed exclusively to man and apes, Tsai designed an experiment in which the rats learned to pull up a bucket paw over paw—an action absent from its natural repertoire (Fig. 5). Realizing that the actions required to achieve the goal were too short, he designed a test demanding several independent actions, of which only the last was rewarded, such as using a tool ladder or pushing a toy car. The rats’ surprisingly high complex-problem-solving speed proved that their actions were not limited to fixed, instinctive patterns. Moreover, the results clearly indicated that rats are as capable of reasoning as humans (Tsai, 1952)—something that Tsai intended to further explore experimentally while avoiding the possibility of practice or trial and error present in previous experiments. He found that the rat seemed able to grasp an abstract idea—having learned to reach food through a door marked with two images, as opposed to one or three. The pictures (fluctuating between faces and other images) were constantly rotated; regardless of where (right, left, or center) they appeared and what they depicted, the rat always picked the door with two images on it and accessed its dinner, offering conclusive evidence of lower animals’ reasoning ability (Fig. 6). Tsai proposed that this reflects the first distinguishing quality of reason: generalizing and reasoning from an abstract idea, labeling it the idea of “two-ness” (Tsai, 1954). The Tsai’s profound insights into animal behavior and original experimental evidence were a basis for further in-depth studies of the behavioral changes of rats from the social competition perspective. From 1953 to 1970, Tsai published research on the competitive behavior of rats, included a) the dominance hierarchy and time gradients (Tsai, 1953), b) social conditioning in the change of dominance hierarchy in white rats (Tsai & Napier, 1968), and c) strengthening the reinforcing displaced aggression and dominance hierarchy in white rats (Tsai & Dexter, 1970). Moreover, because of his pioneering work in describing competitive behavior in the so called “dominance tube”, the instrument used in the experiment was named “Tsai tube” after him (Timmermans, 1978).

Following a remarkable career in experimental psychology, Dr. Tsai moved to the Psychology Department of California State University’s Fullerton Campus in 1965, where he provided leadership and purpose, in a warm and diplomatic way, to a department in its fledgling stages. Students revered his teaching capabilities—he received a distinguished teaching award in 1969 and was named CSUF outstanding Professor in 1971. Despite his retirement in 1973, Tsai remained active in psychology, publishing important experimental research results in his later years, including a) the relative effects of scopolamine and electroconvulsive shock on habit reversal in white rats (Tsai, 1987a), b) the influence of various tridimensional cues on solving a tetrahedron problem (Tsai, 1987b), c) overt and covert problem solving, transfer effects, and programming sequence (Tsai, 1987c), d) the cognitive maze (Tsai, 1987d), and e) the champagne glass illusion (Tsai, 1988). Aged 91 years, he passed away due to a heart attack on December 31st, 1992. In a testimony to Tsai’s legacy, Professor Richard H. Lindley commented:

“The theme of Dr. Tsai’s professional life was a strong unwavering belief that the empirical method of science could be applied to psychological problems.

Dr. Tsai was a brilliant and ingenious researcher and, in many diverse empirical studies, illustrated how basic psychological experimentation could be applied to everyday life and to the most pressing societal concerns.”
